# Factors associated with media use for parenting information: A cross‐sectional study among parents of children aged 0–8 years

**DOI:** 10.1002/nop2.1084

**Published:** 2021-10-21

**Authors:** Irene N. Fierloos, Dafna A. Windhorst, Yuan Fang, Yuping Mao, Matty R. Crone, Clemens M.H. Hosman, Wilma Jansen, Hein Raat

**Affiliations:** ^1^ Department of Public Health Erasmus University Medical Center Rotterdam The Netherlands; ^2^ Department of Cognitive Neuroscience Donders Institute for Brain Cognition and Behaviour Radboud University Medical Center Nijmegen The Netherlands; ^3^ TNO Child Health Leiden The Netherlands; ^4^ Department of Communication Studies California State University Long Beach Long Beach CA USA; ^5^ Department of Public Health and Primary Care Leiden University Medical Center Leiden The Netherlands; ^6^ Department of Health Promotion Maastricht University Maastricht The Netherlands; ^7^ Department of Clinical Psychology Radboud University Nijmegen The Netherlands; ^8^ Hosman Prevention and Innovation Consultancy Berg en Dal The Netherlands; ^9^ Department of Social Development Municipality of Rotterdam Rotterdam The Netherlands

**Keywords:** information seeking, Internet, media, nursing, parenting support

## Abstract

**Aim:**

Media use may strengthen parents’ capacities to deal with parenting issues. This study examined which factors are associated with media use for parenting information.

**Design:**

Cross‐sectional data of 658 parents of children aged 0–8 years, gathered in the CIKEO cohort study in the Netherlands, were analysed.

**Methods:**

Multivariable logistic regression models were used to examine which factors were associated with media use for parenting information.

**Results:**

The mean age of the participants was 33.8 years (*SD* = 5.0); 94.7% were mothers; 77.4% used media for parenting information. Parents with more questions or concerns (OR: 1.40, 95% CI: 1.23, 1.59), and parents who received parenting information from their social contacts (OR: 5.57, 95% CI: 3.22, 9.61), had higher odds of media use for parenting information. Older parents (OR: 0.95, 95% CI: 0.91, 1.00), and parents of older children (OR: 0.84, 95% CI: 0.74, 0.95), had lower odds of media use for parenting information.

## 1 INTRODUCTION

The majority of parents have questions or concerns about their child's health, behaviour, development or their own parenting skills (Reijneveld et al., 2008). These questions or concerns are often interpreted as problems that require the help of a professional (Kesselring et al., 2012), which may be related to the rising demand for specialized youth and family care, including youth mental health care and intensive parenting support (Daly & Bray, 2015; Hilderink et al., 2020; Olfson et al., 2015; Wiens et al., 2020). Recently, there has been increased attention for policies that strengthen parents’ capacities to deal with parenting issues within their social networks and communities (Daly, 2015; Knijn & Hopman, 2015). This may reduce the burden on specialized youth and family care (Daly, 2015; Knijn & Hopman, 2015).

Information and awareness raising through media is a potentially cost‐efficient strategy to provide large groups of parents with evidence‐based parenting information (Metzler et al., 2012). In previous studies, media use with regard to parenting issues has been associated with improved parenting skills, a higher parenting sense of competence, and decreased feelings of depression, anxiety and stress (Calam et al., 2008; Hudson et al., 2003; Kaplan et al., 2014; Na & Chia, 2008; Nieuwboer et al., 2013a, 2013b). Obtaining evidence‐based parenting information may motivate parents to improve their parenting skills and support them to nurture their child's health and well‐being (Calam et al., 2008; Nieuwboer et al., 2013a). However, the quality of parenting information available by media varies (Pehora et al., 2015). According to Khoo et al. (2008), the majority of parents would appreciate more guidance on the quality of parenting information provided by media, preferably from a doctor or a nurse (Khoo et al., 2008).

## 2 BACKGROUND

Many parents use parenting information provided by books, magazines, television, radio and the Internet (Radey & Randolph, 2009). In particular, the use of digital information rapidly increased in the past decades (Lupton et al., 2016; Nieuwboer et al., 2013b). The majority of parents use parenting websites and discussion forums (Baker et al., 2017; Radey & Randolph, 2009; Rothbaum et al., 2008), and nearly half of the parents seek or exchange parenting information through social media, such as Twitter, Facebook, YouTube, Pinterest and Instagram (Baker et al., 2017; Lupton et al., 2016). Lambert and Loiselle (2007) suggest that a parent's decision to seek information is influenced by need, personal and contextual factors. Need factors relate to a perceived gap between what a parent knows and wants to know (Boot & Meijman, 2010; Lambert & Loiselle, 2007). Personal factors relate to socio‐demographic characteristics and psychosocial characteristics, such as personality traits, skills and attitudes with regard to information seeking (Lambert & Loiselle, 2007; Longo, 2005). Contextual factors relate to the broader information environment and context, such as the accessibility of information, and information provided by family and friends (Lambert & Loiselle, 2007; Longo, 2005).

Few previous studies have examined which need and contextual factors are associated with parents’ media use for parenting information. Various studies have examined personal factors (Baker et al., 2017; Malone et al., 2014; Radey & Randolph, 2009; Rothbaum et al., 2008; Sarkadi & Bremberg, 2005; Stern et al., 2012; Walker, 2005). Some studies indicate that several groups of parents, including parents with a low socioeconomic position, may less often use media for parenting information, but the findings are inconsistent (Baker et al., 2017; Malone et al., 2014; Radey & Randolph, 2009; Rothbaum et al., 2008; Sarkadi & Bremberg, 2005; Stern et al., 2012; Walker, 2005).

Gaining more insight into factors associated with media use for parenting information is considered to be a crucial step towards developing strategies that foster the use of evidence‐based parenting information (Metzler et al., 2012; Pehora et al., 2015). This study answers the question: “Which need, personal, and contextual factors are associated with media use for parenting information among parents of children aged 0–8 years?“. We distinguish between various sources of online and offline media, using data from a large community‐based sample of parents (Windhorst et al., 2019).

## 3 THE STUDY

### 3.1 Design

Data for the current study were obtained from an observational cohort study that was embedded in the Consortium Integration Knowledge promotion Effectiveness of parenting interventions (CIKEO; Windhorst et al., 2019). The CIKEO cohort study was designed as a naturalistic effect evaluation, to investigate associations between the use of (elements of) various types of parenting support and outcomes about parenting and child development (Windhorst et al., 2019). The target population were parents/ caregivers of children aged 0–8 years who were living in the Netherlands. Participants were enrolled between October 2017 and December 2019, in two parts. Two preventive Youth Health Care providers in the area of Rotterdam and Dordrecht sent invitation letters to 6,506 parents/caregivers of a child aged 15 months to 6 years in their registry (Part A). Parents/ caregivers with multiple children in this age range could participate with one of their children; the name of this child was mentioned in the invitation letter. In addition, parents/caregivers of children aged 0–8 years who were planning to participate in parenting intervention programmes were recruited via providers of parenting intervention programmes across the Netherlands and directly via advertisements on websites, discussion forums and Facebook pages related to parenting (Part B). For the purpose of the current study, we used data of Part A.

All invited families received an informed consent form, a baseline questionnaire and an information letter with the request whether the parent/ caregiver who spends most time with the child would complete the questionnaire. Parents/ caregivers could participate voluntarily by returning the informed consent form and the questionnaire to the researchers in a pre‐paid envelope or via the Internet. All parents/caregivers who provided written informed consent and a completed questionnaire were enrolled in the study. After 12 months, participants received a follow‐up questionnaire by post or via the Internet, depending on their preference.

**FIGURE 1:**
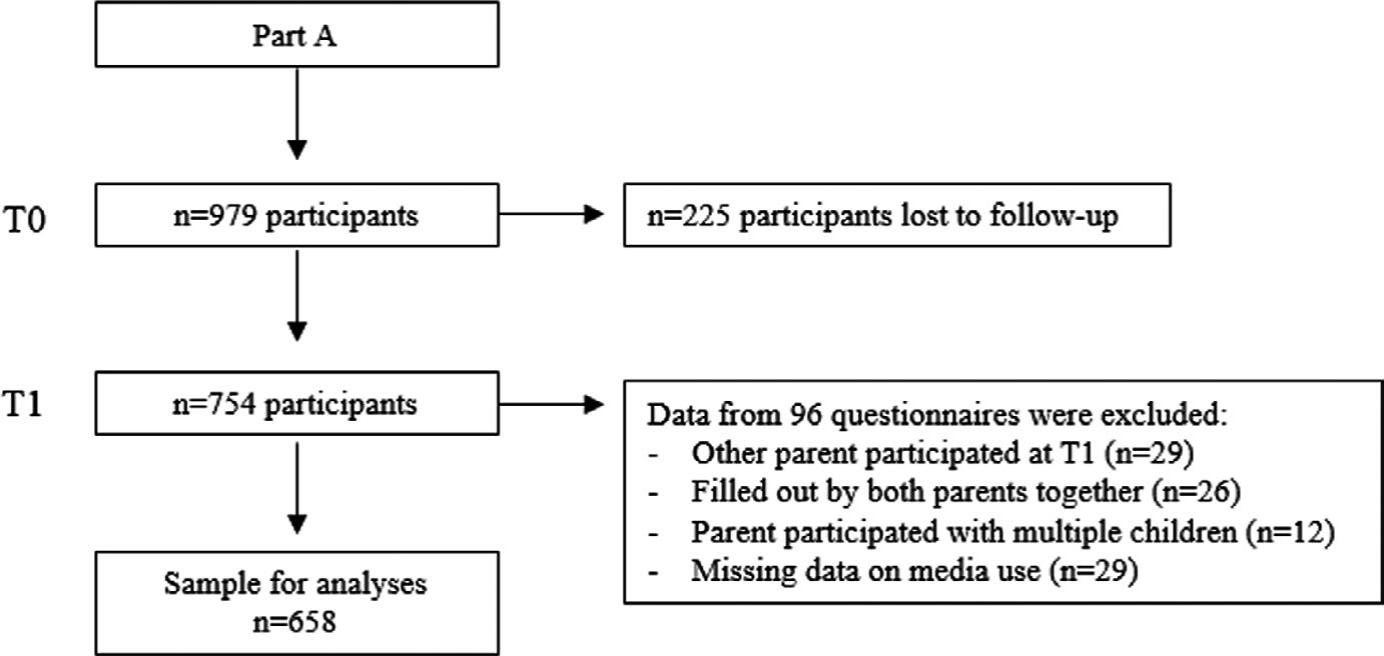
Flow chart of the inclusion process of the CIKEO cohort study and the sample for analyses (*n* = 658)

In total, 979 parents in Part A participated in the baseline measurement (Figure 1); 225 parents were lost to follow‐up; data from 29 questionnaires were excluded because the follow‐up questionnaire was not filled out by the same parent; data from 26 questionnaires completed by two parents together were excluded from the analyses; 12 parents participated in the study with multiple children, and data from their second questionnaires were excluded from the analyses. In addition, data from 29 questionnaires were excluded due to missing information on the outcome of interest. Hence, the population for analyses consisted of 658 participants.

## 4 METHOD

### 4.1 Media use for parenting information

The use of parenting information was assessed in the follow‐up questionnaire, by a series of questions that asked whether the parent used parenting information provided by (1) websites, (2) discussion forums, (3) social media (e.g. Twitter, Facebook, Instagram and YouTube), (4) WhatsApp chat groups, (5) (digital) magazines, (6) books and (7) personal social contacts (family, friends, acquaintances, neighbours or colleagues). The answer options were “often,” “sometimes” and “never” and were recoded into a dichotomous variable indicating whether this source of parenting information was used. “Often” and “sometimes” were recoded into “yes,” and “never” was recoded into “no.” Media were defined as channels or systems of communication or information (Merriam‐Webster, n.d.). Information provided by personal social contacts did not fit the definition of media, as it refers to a direct exchange of information. Three variables (yes/no) were created to indicate overall media use (1–6), online media use (1–4) and offline media use (6).

### 4.2 Need, contextual and personal factors

The number of questions or concerns about topics related to parenting was studied as a need factor (Boot & Meijman, 2010). Parental questions or concerns were assessed in the follow‐up questionnaire by a list of 21 frequent parenting issues (Table 1; Oudhof et al., 2013). Parents were asked to indicate the issues on which they have had questions or concerns in the 12 months prior to the follow‐up measurement. Other issues could be specified in an open text box. The 21 topics were divided into the following themes: “parenting,” “child development,” “sleeping,” “food,” “child behaviour and emotions” and “media use of the child” (Table 1).

**TABLE 1:** Questions or concerns related to parenting issues in the 12 months prior to the follow‐up measurement of the CIKEO study (*n* = 658)

Category	Topics	Number of parents reporting questions or concerns *n* (% of total)
Parenting	Setting rules and limits	151 (22.9%)
	Punishing and rewarding	121 (18.4%)
	Functioning as a parent	80 (12.2%)
	Communication parent–child	79 (12.0%)
Child development	Becoming potty‐trained	134 (20.4%)
	Speech and language development	82 (12.5%)
	School performance	48 (7.3%)
	Motor development and movement	39 (5.9%)
	Physical development	23 (3.5%)
Sleeping	Sleeping	104 (15.8%)
Food	Food	102 (15.5%)
Child behaviour and emotions	Listening, obeying	118 (17.9%)
	Temper tantrums, anger, aggression	103 (15.7%)
	Social contact	46 (7.0%)
	Fear, insecurity	43 (6.5%)
	Dealing with changes	39 (5.9%)
	Sad, crying a lot	32 (4.9%)
	Fantasies or dreams	24 (3.6%)
	Bullying, being bullied	21 (3.2%)
Media use child	Media use child	33 (5.0%)

Personal factors were assessed in the baseline questionnaire. Personal factors concerning the responding parent/ caregiver were age (in years), gender (male/ female), educational level, employment status and migration background. Educational level was assessed by the highest completed education and was reclassified into three categories based on the 2011 International Standard Classification of Education (ISCED). ISCED level 0–2 (no education, primary education, lower secondary education) was categorized as “low”; ISCED level 3–5 (upper secondary education, postsecondary non‐tertiary education, short‐cycle tertiary education) was categorized as “middle”; ISCED level 6–8 (bachelor, master, doctoral or equivalent) was categorized as “high” (Statistics, 2012). Parents were asked to specify their employment status as: “working fulltime,” “working part‐time,” “stay‐at‐home parent,” “unemployed,” “incapacitated,” “studying” and “other.” Working fulltime and part‐time were categorized as “paid job”; the remaining categories were categorized as “no paid job.” Migration background was assessed by country of birth. When either the responding parent or one or both of his/ her parents were born outside the Netherlands, this was categorized as a migration background (CBS, 2016). The following personal factors with regard to the child and family were studied: age of the child (in years), gender of the child (boy/ girl), number of children in the household (one/ two/ more than two) and family composition (one‐parent family/ two‐parent family).

Parenting information provided by personal social contacts (yes/ no), as described earlier (question 7 on the use of parenting information), was studied as a contextual factor.

### 4.3 Analysis

Descriptive statistics were used to characterize the participants, to describe the frequency of media use and to describe the topics on which parents had questions or concerns. Full multivariable logistic regression models were used to assess independent associations between need, personal and contextual factors, and the use of (1) overall media, (2) online media, (3) offline media, (4) websites, (5) discussion forums, (6) social media, (7) WhatsApp chat groups and 8) (digital) magazines for parenting information. Odds ratios (OR) and 95% confidence intervals (95% CI) were calculated for each factor.

To assess moderating effects, interaction terms were separately added to the full regression models on media use for parenting information. A Bonferroni correction for multiple testing was applied (*p* = .05/30 = 0.002). No statistically significant interactions were found. *p*‐values of the interaction analyses are presented in Table S1.

Multiple imputation was used to handle missing values of the need, personal and contextual factors. Missing values varied between 0.2% (*n* = 1) for gender of the child and 0.6% (*n* = 4) for age of the child (Table 2). Five imputed data sets were created for pooled estimates. The regression analyses were performed in both the non‐imputed and the imputed data set, and the results were similar (data not shown). Data were analysed in Statistical Package for Social Sciences, version 25 for Windows (IBM SPSS Statistics for Windows, IBM Corp). *p*‐values below .05 were considered statistically significant.

**TABLE 2:** Characteristics of 658 parents of children aged 0–8 years participating in the CIKEO study; by media use for parenting information

	Total	Media use for parenting information: yes	Media use for parenting information: no	
	*n* = 658	*n* = 509 (77.4%)	*n* = 149 (22.6%)	
	mean (*SD*) *n* (%)	mean (*SD*) *n* (%)	mean (*SD*) *n* (%)	*p*‐value
**Need factors**				
*Number of questions or concerns related to parenting issues*	2.4 (*SD* = 2.4)	2.7 (*SD* = 2.5)	1.3 (*SD* = 1.7)	**<.001**
**Personal factors**				
*Age of the parent (in years)*	33.8 (*SD* = 5.0)	33.2 (*SD* = 4.7)	35.8 (*SD* = 5.2)	**<.001**
*Gender of the parent*				.091
Female	623 (94.7%)	486 (95.5%)	137 (91.7%)	
Male	35 (5.3%)	23 (4.5%)	12 (8.1%)	
*Educational level of the parent* ^a^				
High	371 (56.4%)	286 (56.3%)	85 (57.0%)	.055
Middle	249 (37.8%)	199 (39.2%)	50 (33.6%)	
Low	37 (5.6%)	23 (4.5%)	14 (9.4%)	
*Employment status of the parent*				
Paid job	542 (82.4%)	421 (82.9%)	121 (81.8%)	.752
No paid job	114 (17.3%)	87 (17.1%)	27 (18.2%)	
*Migration background of the parent*				
No	583 (88.6%)	452 (88.8%)	131 (87.9%)	.766
Yes	75 (11.4%)	57 (11.2%)	18 (12.1%)	
*Family situation*				
Two‐parent family	626 (95.1%)	489 (96.1%)	137 (91.9%)	.**004**
One‐parent family	32 (4.9%)	20 (3.9%)	12 (8.1%)	
*Age of the child (in years)*	3.2 (*SD* = 1.9)	3.0 (*SD* = 1.8)	3.9 (*SD* = 1.9)	**<.001**
*Gender of the child*				
Girl	318 (48.3%)	255 (50.1%)	63 (42.6%)	.107
Boy	339 (51.5%)	254 (49.9%)	85 (57.4%)	
*Number of children in the household*				
One child	195 (29.6%)	161 (31.6%)	34 (22.8%)	.116
Two children	292 (44.4%)	219 (43.0%)	73 (49.0%)	
More than two children	171 (26.0%)	129 (25.3%)	42 (28.2%)	
**Contextual factors**				
*Parenting information from personal social contacts*				
No	80 (12.2%)	31 (6.1%)	49 (32.9%)	**<.001**
Yes	587 (87.8%)	478 (93.9%)	100 (67.1%)	

Note

*p*‐values <.05 in bold. *p*‐values for continuous variables were calculated with independent *t* tests, and *p*‐values for categorical variables were calculated with chi‐squared tests. Missing values: educational level *n* = 1; employment status: *n* = 2; age child *n* = 4; gender child *n* = 1.

Abbreviations: *SD*= standard deviation.

^a^ Educational level “High”: bachelor, master, doctoral or equivalent; “Middle”: upper secondary education, postsecondary non‐tertiary education, short‐cycle tertiary education; “Low”: no education, primary education, lower secondary education.

### 4.4 Non‐response analysis

The socio‐demographic characteristics of participants who were excluded from the sample for analyses due to missing data or lost to follow‐up (*n* = 321) were compared with the characteristics of participants in the sample for analyses (*n* = 658) using *t* tests and chi‐squared tests. Compared to participants in the sample for analyses, excluded participants were more often fathers (*p* < .001), more often had a lower educational level (*p* = .006), and more often had a migration background (*p* = .048). No other differences were found (*p* > .05).

### 4.5 Ethics

The Medical Ethics Committee of the Erasmus Medical Center, Rotterdam, decided that the rules laid down in the Dutch Medical Research Involving Human Subjects Act (in Dutch: “Wet Medisch‐wetenschappelijk Onderzoek met mensen”) did not apply to the research proposal (proposal number MEC‐2017‐432). The CIKEO cohort study was registered as NL7342 in the Netherlands Trial Registry (Windhorst et al., 2019).

## 5 RESULTS

### 5.1 Questions or concerns about topics related to parenting

Table 1 presents the topics on which parents had questions or concerns. Frequent questions or concerns related to parenting were about setting rules and limits (22.9%) and punishing or rewarding (18.4%). Frequent questions or concerns about the child were about becoming potty‐trained (20.4%) and listening or obeying (17.9%).

### 5.2 Characteristics of the participants

Table 2 presents the characteristics of the participants. The mean age of the responding parents was 33.8 (*SD* = 5.0) years. In total, 94.7% of the responding parents were women. The majority of the responding parents had a high educational level (56.4%), a paid job (82.4%) and no migration background (88.6%). The mean age of the child was 3.2 (*SD* = 1.9) years.

About three quarters of the responding parents (77.4%) used media for parenting information; 27.5% of the parents used both online and offline media; 41.2% of the parents only used online media; 4.7% of the parents only used offline media. Table 3 presents the frequency of specific types of media use for parenting information. Parenting websites were used most frequently.

**TABLE 3:** Frequency of media use for parenting information among participants of the CIKEO study (*n* = 658)

	“Often” used for parenting information	“Sometimes” used for parenting information	“Never” used for parenting information
	*n* (% of total)	*n* (% of total)	*n* (% of total)
Parenting websites	41 (6.2%)	366 (55.6%)	251 (38.1%)
Discussion forums	13 (2.0%)	177 (26.9%)	468 (71.1%)
Social media	18 (2.7%)	151 (22.9%)	489 (74.3%)
WhatsApp chat groups	16 (2.4%)	63 (9.6%)	579 (88.0%)
(Digital) magazines	44 (6.7%)	290 (44.1%)	324 (49.2%)
Books	23 (3.5%)	189 (28.7%)	446 (67.8%)

### 5.3 Media use for parenting information

Table 4 presents the fully adjusted regression models on the associations between need, personal, and contextual factors and media use for parenting information. The fully adjusted regression model for overall media use showed that parents with more questions or concerns related to parenting issues (OR: 1.40, 95% CI: 1.23, 1.59), and parents who received parenting information from their personal social contacts (OR: 5.57, 95% CI: 3.22, 9.61), had higher odds of media use for parenting information. Older parents (OR: 0.95, 95% CI: 0.91, 1.00), and parents of older children (OR: 0.84, 95% CI: 0.74, 0.95), had lower odds of media use for parenting information.

**TABLE 4:** Associations between need, personal, and contextual factors and media use for parenting information among participants of the CIKEO study (*n* = 658)

	Overall media use (“yes” *n* = 509; 77.4%)	Online media use (“yes” *n* = 452; 68.7%)	Offline media use (“yes” *n* = 212; 32.2%)
	Full model	Full model	Full model
	OR (95% CI)	OR (95% CI)	OR (95% CI)
**Need factors**			
*Questions or concerns related to parenting issues (more)*	1.40 (1.23, 1.59)***	1.38 (1.24, 1.54)***	1.18 (1.09, 1.27)***
**Personal factors**			
*Age of the parent (in years)*	0.95 (0.91, 1.00)*	0.93 (0.89, 0.98)**	1.01 (0.97, 1.05)
*Gender of the parent*			
Female	ref.	ref.	ref.
Male	1.32 (0.52, 3.38)	1.36 (0.56, 3.30)	1.16 (0.53, 2.58)
*Educational level of the parent* ^a^			
High	ref.	ref.	ref.
Middle	1.14 (0.72, 1.81)	1.09 (0.72, 1.64)	0.63 (0.44, 0.92)*
Low	0.73 (0.32, 1.63)	0.73 (0.33, 1.58)	0.51 (0.21, 1.23)
*Employment status of the parent*			
Paid job	ref.	ref.	ref.
No paid job	0.83 (0.48, 1.45)	0.73 (0.44, 1.20)	1.09 (0.69, 1.73)
*Migration background of the parent*			
No	ref.	ref.	ref.
Yes	1.20 (0.63, 2.31)	1.43 (0.78, 2.63)	1.07 (0.62, 1.85)
*Family situation*			
Two‐parent family	ref.	ref.	ref.
One‐parent family	0.64 (0.26, 1.60)	0.78 (0.32, 1.86)	0.85 (0.34, 2.12)
*Age of the child (in years)*	0.84 (0.74, 0.95)**	0.87 (0.78, 0.97)*	0.97 (0.88, 1.08)
*Gender of the child*			
Girl	ref.	ref.	ref.
Boy	0.68 (0.44, 1.03)	0.62 (0.42, 0.91)*	0.72 (0.51, 1.02)
*Number of children in the household*			
One child	ref.	ref.	ref.
Two children	0.78 (0.45, 1.35)	0.94 (0.58, 1.54)	1.27 (0.82, 1.98)
More than two children	1.15 (0.61, 2.18)	1.08 (0.61, 1.90)	1.85 (1.10, 3.12)*
**Contextual factors**			
*Parenting information from personal social contacts*			
No	ref.	ref.	ref.
Yes	5.57 (3.22, 9.61)***	5.09 (2.90–8.92)***	2.01 (1.07, 3.79)*

Note

Odds ratios and 95% confidence intervals were derived from the logistic regression analyses for overall, online and offline media use for parenting information. *p*‐values <.05 in bold.

Abbreviations: CI= confidence interval; OR= odds ratio; ref.= reference group.

*p‐value <.05, ** *p*‐value <.01 and ***p‐value <.001.

^a^ Educational level “High”: bachelor, master, doctoral or equivalent; “Middle”: upper secondary education, postsecondary non‐tertiary education, short‐cycle tertiary education; “Low”: no education, primary education, lower secondary education.

The fully adjusted regression model for online media use (Table 4) shows that parents with more questions or concerns related to parenting issues (OR: 1.38, 95% CI: 1.24, 1.54), and parents who received parenting information from their personal social contacts (OR: 5.09, 95% CI: 2.90–8.92), had higher odds of online media use for parenting information. Older parents (OR: 0.93, 95% CI: 0.89, 0.98), parents of older children (OR: 0.87, 95% CI: 0.78, 0.97) and parents of boys (OR: 0.62, 95% CI: 0.42, 0.91) had lower odds of online media use for parenting information.

The fully adjusted regression model for offline media use (Table 4) shows that parents with more questions or concerns related to parenting issues (OR: 1.18, 95% CI: 1.09, 1.27), parents of more than two children (OR: 1.85, 95% CI: 1.10, 3.12) and parents who received parenting information from their personal social contacts (OR: 2.01, 95% CI: 1.07, 3.79) had higher odds of offline media use for parenting information. Compared to parents with a high educational level, parents with a middle educational level (OR: 0.63, 95% CI: 0.44, 0.92) had lower odds of offline media use for parenting information.

Table 5 presents the fully adjusted regression models for the use of “websites,” “discussion forums,” “social media,” “WhatsApp chat groups” and “(digital) magazines.” Having more questions or concerns related to parenting issues was associated with higher odds of all types of media use (*p* < .05), except (digital) magazines (OR: 1.07, 95% CI: 0.99, 1.15). All personal factors, except migration background, were associated with one or more specific types of media use (*p* < .05). Receiving parenting information from personal social contacts was associated with higher odds of all types of media use (*p* < .05), except WhatsApp chat groups (OR: 2.04, 95% CI: 0.70, 5.98).

**TABLE 5:** Associations between need, personal, and contextual factors and the use of specific types of media for parenting information among participants of the CIKEO study (*n* = 658)

	Websites (“yes” *n* = 407; 61.9%)	Discussion forums (“yes” *n* = 190; 28.9%)	Social media (“yes” *n* = 169; 25.7%)	WhatsApp (“yes” *n* = 79; 12.0%)	(Digital) magazines (“yes” *n* = 334; 50.8%)
	Full model	Full model	Full model	Full model	Full model
	OR (95% CI)	OR (95% CI)	OR (95% CI)	OR (95% CI)	OR (95% CI)
**Need factors**					
*Questions or concerns related to parenting issues (more)*	1.29 (1.18, 1.42)***	1.17 (1.08, 1.26)***	1.23 (1.14, 1.33)***	1.17 (1.12, 1.23)***	1.07 (0.99, 1.15)
**Personal factors**					
*Age of the parent (in years)*	0.93 (0.89, 0.97)**	0.93 (0.89, 0.98)**	0.96 (0.92, 1.01)	0.97 (0.91, 1.03)	0.96 (0.93, 1.00)*
*Gender of the parent*					
Female	ref.	ref.	ref.	ref.	ref.
Male	1.52 (0.65, 3.56)	1.09 (0.44, 2.70)	0.12 (0.02, 0.92)*	1.46 (0.46, 4.69)	0.47 (0.21, 1.09)
*Educational level of the parent* ^a^					
High	ref.	ref.	ref.		ref.
Middle	0.78 (0.53, 1.14)	0.83 (0.56, 1.23)	1.54 (1.03, 2.28)*	1.59 (0.95, 2.68)	1.05 (0.73, 1.50)
Low	0.72 (0.33, 1.54)	1.08 (0.46, 2.51)	1.07 (0.42, 2.70)	0.67 (0.15, 3.03)	0.55 (0.25, 1.22)
*Employment status of the parent*					
Paid job	ref.	ref.	ref.	ref.	ref.
No paid job	0.56 (0.35, 0.89)*	1.16 (0.70, 1.93)	1.12 (0.67, 1.86)	0.95 (0.48, 1.90)	0.62 (0.39, 0.97)*
*Migration background of the parent*					
No	ref.	ref.	ref.	ref.	ref.
Yes	1.67 (0.93, 3.00)	1.00 (0.55, 1.83)	0.99 (0.54, 1.83)	1.45 (0.69, 3.06)	1.58 (0.92, 2.72)
*Family situation*					
Two‐parent family	ref.	ref.	ref.	ref.	ref.
One‐parent family	0.90 (0.39, 2.08)	0.64 (0.24, 1.73)	1.82 (0.78, 4.28)	0.87 (0.48, 1.57)	0.28 (0.11, 0.71)**
*Age of the child (in years)*	0.88 (0.79, 0.98)*	0.90 (0.80, 1.01)	1.07 (0.95, 1.20)	1.06 (0.91, 1.24)	0.88 (0.80, 0.98)*
*Gender of the child*					
Girl	ref.	ref.	ref.	ref.	ref.
Boy	0.71 (0.49, 1.01)	0.68 (0.47, 0.98)*	0.78 (0.53, 1.15)	0.57 (0.35, 0.94)*	0.96 (0.68, 1.33)
*Number of children in the household*					
One child	ref.	ref.	ref.	ref.	ref.
Two children	0.97 (0.61, 1.53)	0.67 (0.43, 1.03)	0.98 (0.61, 1.55)	1.07 (0.60, 1.90)	0.62 (0.41, 0.95)*
More than two children	1.06 (0.62, 1.80)	0.56 (0.32, 0.97)*	0.85 (0.47, 1.52)	0.45 (0.20, 1.05)	0.64 (0.39, 1.06)
**Contextual factors**					
*Parenting information by personal social contacts*					
No	ref.	ref.	ref.	ref.	ref.
Yes	4.83 (2.70, 8.65)***	3.39 (1.49, 7.73)**	4.08 (2.51, 6.61)**	2.04 (0.70, 5.98)	3.51 (1.94, 6.36)***

Note

Odds ratios and 95% confidence intervals were derived from the multivariable logistic regression analyses for specific types of media use for parenting information. *p*‐values <.05 in bold. Abbreviations: OR= odds ratio; CI= confidence interval; ref.= reference group.

*p‐value <.05, **p‐value <.01 and ***p‐value <.001.

^a^ Educational level “High”: bachelor, master, doctoral or equivalent; “Middle”: upper secondary education, postsecondary non‐tertiary education, short‐cycle tertiary education; “Low”: no education, primary education, lower secondary education.

## 6 DISCUSSION

This study examined which need, personal and contextual factors are associated with media use for parenting information by parents of children aged 0–8 years. Parents with more questions or concerns related to parenting issues (need factor), younger parents, and parents of younger children (personal factors), and parents who received parenting information from personal social contacts (contextual factor) had higher odds of overall media use for parenting information. All other personal factors, except migration background, were associated with one or more specific types of media use.

### 6.1 Need factors

Having more questions or concerns related to parenting issues was independently associated with higher odds of overall, online and offline media use for parenting information. This is in line with previous studies in which parental concerns have been associated with formal help‐seeking (Ellingson et al., 2004; Farmer et al., 1999). Having questions or concerns may evoke information seeking in order to gain factual information and to reduce feelings of uncertainty (Boot & Meijman, 2010). At the same time, obtaining parenting information may also raise new questions or concerns (Rathbone & Prescott, 2019). Longitudinal research is needed to gain more insight into the directions of this association.

Many parents had questions or concerns about setting rules and limits, and punishing and rewarding. Frequent themes about the child were becoming potty‐trained and listening and obeying. In additional analyses, we explored whether the specific themes on which parents had questions or concerns were associated with overall, online and offline media use. The results of the full multivariable logistic regression models are presented in Table S2. The specific themes on which parents had questions or concerns were not associated with overall media use, which indicates that parents are using media for a broad variety of parenting issues.

### 6.2 Personal factors

The age of the parent and the age of the child were independently associated with overall and online media use for parenting information, with lower odds of media use when age increases, which is in line with previous findings (Baker et al., 2017; Radey & Randolph, 2009; Sarkadi & Bremberg, 2005). This study showed that these associations remain significant after adjusting for parental questions or concerns. We hypothesize that younger parents may have been using the Internet from a younger age, which perhaps made them more familiar with the use of online media for support (Baker et al., 2017). We suggest that media use for parenting information may be particularly important for parents of younger children, as it offers experience‐based peer support and assistance for specific issues related to child development in the first years (Bernhardt & Felter, 2004; Plantin & Daneback, 2009).

The gender of the parent was not independently associated with overall, online and offline media use for parenting information. Previous studies found that fathers less often used media with regard to parenting issues (Baker et al., 2017; Radey & Randolph, 2009; Sarkadi & Bremberg, 2005; Stern et al., 2012). The number of fathers in our sample was low, which may have resulted in a lack of statistical power to evaluate gender differences. It has been suggested that, in general, fathers are less likely to be the primary caregiver of the child and might thereby less often seek parenting information (Baker et al., 2017; Metzler et al., 2012; Radey & Randolph, 2009). In addition, Sarkadi and Bremberg (2005) have suggested that online media, including discussion platforms, may be tailored towards mothers (Sarkadi & Bremberg, 2005). Future studies on media use for parenting issues should pay specific attention to the inclusion of a large and diverse group of fathers.

The educational level of the parent was not independently associated with overall and online media use for parenting information. However, parents with a lower educational levels had lower odds of using offline media for parenting information, compared to parents with a high educational level. The results of previous studies were inconsistent (Baker et al., 2017; Guillory et al., 2014; Radey & Randolph, 2009; Rothbaum et al., 2008; Sarkadi & Bremberg, 2005). There may be educational disparities due to differences in health literacy: the cognitive and social skills that influence a parent's motivation and ability to access, understand and use parenting information (Nutbeam, 2009; Rothbaum et al., 2008; Van Dijk, 2006). Parents with different socioeconomic positions and/ or educational levels may differ in their media preference and their evaluation of the trustworthiness of these sources (Malone et al., 2014; Rothbaum et al., 2008). The results of this study indicate there may be less educational disparities for online media than for offline media. On the one hand, this is promising as online media may provide means to reach a larger and more diverse group of parents with parenting information. On the other hand, this may raise concerns, because the quality of the parenting information provided by social media and discussion forums may be lower, and the information may sometimes be inaccurate (Chung et al., 2012).

Additional analyses were conducted to explore the association between educational level and information triangulation: the use of both online and offline media (Table S3). Parents with a low and a middle educational level had significantly lower odds of using a combination of online and offline sources, compared to parents with a high educational level. This may be disadvantageous, as obtaining information from multiple sources may foster a more critical evaluation of the accuracy of the gathered information (O'Connor & Madge, 2004). We also explored whether household income was independently associated with media use, and this was not the case (*p*‐values of the association in full multivariable logistic regression models for overall media use, online media use and offline media use >.05 (data not shown)).

Having a migration background was not independently associated with media use for parenting information. This is in line with previous findings (Radey & Randolph, 2009). We hypothesize that media are an important source of parenting information to parents with a migration background, as their access to formal care may be decreased due to language and/ or cultural differences (Hernández‐plaza et al., 2004). Media may offer parenting information in their first language (Hernández‐plaza et al., 2004; Mao, 2015).

### 6.3 Contextual factors

Parents receiving parenting information from personal social contacts had higher odds of media use for parenting information, independent of their need for parenting information. This is in line with results of previous studies which showed that social contacts influence information seeking by referring to information sources, by supporting the retrieval of information and by discussing and evaluating information (Boot & Meijman, 2010; Brashers et al., 2002; Walker & Riley, 2001). We hypothesize that receiving parenting information from personal social contacts may increase parents’ motivation to seek and exchange parenting information (Boot & Meijman, 2010). We recommend the use of qualitative and/ or mixed methods designs to gain more insight into the specific pathways of this association. Future studies should also examine other contextual factors, such as the availability of parenting information in different languages and countries (Brashers et al., 2002).

### 6.4 The intensity of media use

In additional analyses, the intensity of media use for parenting information was taken into account. The intensity of media use was calculated as the sum of the use of websites, discussion forums, social media, WhatsApp, (digital) magazines and books (“never” = 0 / “sometimes” = 1/ “often” = 2; range = 0–12). A linear regression model was used to assess independent associations between need, contextual, and personal factors and overall media use. The associations in the full multivariable linear regression model were similar to the associations in the full multivariable logistic regression model for overall media use (data not shown).

### 6.5 Implications for policy and practice

This study indicates that a large and diverse group of parents is using media for parenting information. Parents differ in their media preferences, and the quality of information may vary between these media (Chung et al., 2012; Pehora et al., 2015). Health and social care professionals may encourage the use of evidence‐based parenting information in various ways. First, by spreading evidence‐based parenting information through media that fit the preferences of parents (Radey & Randolph, 2009). Second, by providing guidance on the retrieval of evidence‐based parenting information by media, for example by an “information prescription” with a list of reliable websites (Khoo et al., 2008). Finally, by developing and implementing intervention strategies that enhance media literacy (Doak et al., 1996; Jeong et al., 2012; Pehora et al., 2015) and by training parents to evaluate the trustworthiness of information (Chung et al., 2012). We advise to pay special attention to groups of parents who may be less likely to use media for parenting information, including parents who receive no parenting information from their personal social contacts.

## 7 LIMITATIONS

Strengths of this study include the availability of data on specific types of media, and the large variety of potentially associated factors. Several limitations should also be considered. First, the representativeness of the sample was reduced due to excluded participants and participants that were lost to follow‐up. Fathers, parents with a lower educational level and parents with a migration background were more likely to be excluded from the sample for analyses. The statistical power to detect associations may have been reduced due to this underrepresentation, but we have no rationale to expect that the direction of the associations has been affected. Second, the cross‐sectional design did not allow to infer causality. Future studies may expand upon the findings using longitudinal designs with large and varied samples of mothers and fathers.

## 8 CONCLUSION

Need, personal and contextual factors are associated with media use for parenting information among parents of children aged 0–8 years. Parents with more questions or concerns about topics related to parenting (need factor) and parents who receive parenting information by personal social contacts (contextual factor) had higher odds of media use for parenting information. Older parents and parents of older children (personal factors) had lower odds of media use for parenting information. Although a large and diverse group of parents is using media for parenting information, parents differ in their media preferences, and the quality of information may vary. Special attention should be paid to groups of parents who are less likely to use media for parenting information, including parents who receive no parenting information from their personal social contacts. Health and social care professionals may encourage the use of evidence‐based parenting information by (1) communicating through media that fit the preferences of parents, (2) by providing guidance on media use and (3) by implementing intervention strategies that improve media literacy. Ultimately, more parents will feel empowered to use media to obtain evidence‐based parenting information, which strengthens their capacity to deal with parenting issues.

## CONFLICT OF INTEREST

No conflict of interest has been declared by the authors.

## AUTHOR CONTRIBUTIONS

IF: data collection, conceptualization, analysis, interpretation of the data and writing the original draft; DW: supervision, data collection, interpretation of the data and critical review; YF: data collection and critical review; YM: conceptualization and critical review; MC, CH and WJ: study design and critical review; HR: supervision, study design, data collection, interpretation of the data and critical review. All authors approved the final version.

## Supporting information

Tables S1–S3

## Data Availability

The data that support the findings of this study are available on request from the corresponding author. The data are not publicly available due to privacy or ethical restrictions.
