# Supplementary material for: Factors associated with media use for parenting information: A cross‐sectional study among parents of children aged 0–8 years
Source: Nurs Open. 2021 Oct 21;9(1):446–57. doi: 10.1002/nop2.1084 (PMC8685885; doi:10.1002/nop2.1084)
Supplement: Supplementary file 1 — Tables S1–S3 [file NOP2-9-446-s001.docx]

## Supplementary material Factors associated with media use for parenting information: A cross-sectional study among parents of children aged 0-8 years

**Table S1** Results of the interaction analyses for factors associated with media use for parenting information among participants of the CIKEO study (n=658).

| **Interaction term** | **P-value of the OR in the full model for overall media use** | **P-value of the OR in the full model for online media use** | **P-value of the OR in the full model for offline media use** |
| --- | --- | --- | --- |
| Age of the parent*parental concerns † | .324 | .156 | .115 |
| Gender of the parent (male) *parental concerns | .469 | .163 | .616 |
| Educational level of the parent*parental concerns | .426 | .465 | .122 |
| No paid employment*parental concerns | .424 | .948 | .179 |
| Migration background parent (yes) *parental concerns | .017 | .087 | .344 |
| One-parent family*parental concerns | .772 | .432 | .577 |
| Age of the child*parental concerns | .681 | .949 | .190 |
| Gender of the child (boy) *parental concerns | .632 | .628 | .182 |
| Number of children*parental concerns | .177 | .554 | .338 |
| Parenting information from personal social contacts*parental concerns | .655 | .234 | .116 |

P-values were derived by separately adding the interaction terms to the full multivariable logistic regression models on media use for parenting information (Table 4).
Abbreviations: OR=odds ratio.
Bonferroni correction for multiple testing (.05/30). P-values <.002 in bold.
† Questions or concerns related to parenting issues.

**Table S2** Associations between need, contextual and personal factors and media use for parenting information among participants of the CIKEO study (n=658); including the topics on which parents had questions or concerns.

|  | **Overall media use**  (‘yes’ n=509; 77.4%) | **Online media use** (‘yes’ n=452; 68.7%) | **Offline media use** (‘yes’ n=212; 32.2%) |
| --- | --- | --- | --- |
|  | Full model OR (95% CI) | Full model OR (95% CI) | Full model OR (95% CI) |
| **Need factors**  *Questions or concerns related to parenting issues (more)*  *Questions or concerns about:*  Parenting  Child development  Sleeping  Food  Child behaviour and emotions  Media use child | **1.38 (1.02, 1.86)***  1.09 (0.55, 2.16) 1.33 (0.72, 2.43) 0.97 (0.44, 2.11) 0.67 (0.33, 1.35) 0.85 (0.42, 1.71)  2.60 (0.53, 12.61) | **1.30 (1.02, 1.66)***  0.84 (0.47, 1.52)  1.58 (0.94, 2.66)  1.40 (0.67, 2.92)  0.67 (0.36, 1.25)  1.23 (0.67, 2.27)  1.31 (0.43, 3.99) | 1.11 (0.96, 1.29)  1.58 (0.99, 2.52)  1.03 (0.68. 1.55)  1.35 (0.80, 2.28)  0.86 (0.53, 1.42)  0.90 (0.57, 1.44)  0.94 (0.43, 2.05) |
| **Personal factors** |  |  |  |
| *Age of the parent (in years)* | **0.95 (0.91, 1.00)*** | **0.94 (0.90, 0.98)**** | 1.00 (0.96, 1.05) |
| *Gender of the parent*  Female  Male | ref. 1.38 (0.53, 3.58) | ref. 1.35 (0.55, 3.33) | ref. 1.23 (0.55, 2.74) |
| *Educational level* *of the parent*†  High  Middle  Low | ref. 1.17 (0.73, 1.86) 0.72 (0.32, 1.63) | ref. 1.13 (0.75, 1.72) 0.69 (0.32, 1.51) | ref. **0.63 (0.43, 0.92)*** 0.50 (0.21, 1.22) |
| *Employment status of the parent*  Paid job  No paid job | ref. 0.85 (0.49, 1.49) | ref. 0.74 (0.45, 1.23) | ref. 1.11 (0.70, 1.77) |
| *Migration background of the parent*  No  Yes | ref. 1.17 (0.60, 2.25) | ref. 1.41 (0.76, 2.62) | ref. 1.05 (0.61, 1.84) |
| *Family situation*  Two-parent family  One-parent family | ref. 0.60 (0.24, 1.51) | ref. 0.73 (0.30, 1.77) | ref. 0.85 (0.34, 2.14) |
| *Age of the child (in years)* | **0.83 (0.73, 0.95)**** | **0.85 (0.76, 0.96)**** | 0.99 (0.89, 1.10) |
| *Gender of the child*  Girl  Boy | ref. **0.65 (0.42, 1.00)*** | ref. **0.60 (0.40, 0.88)**** | ref.  0.71 (0.50, 1.01) |
| *Number of children in the household*  One child  Two children  More than two children | ref. 0.77 (0.44, 1.34)  1.15 (0.60, 2.20) | ref. 1.04 (0.63, 1.73)  1.18 (0.66, 2.10) | ref.  1.23 (0.79, 1.93)  **1.88 (1.11, 3.19)*** |
| **Contextual factors** *Parenting information from personal social contacts*  No  Yes | ref. **5.84 (3.34, 10.21)***** | ref. **5.30 (3.00, 9.38)***** | ref. **2.07 (1.09, 3.93)*** |

Odds ratios and 95% confidence intervals were derived from the logistic regression analyses for overall, online and offline media use for parenting information. P-values <.05 in bold.
Abbreviations: OR=odds ratio; CI=confidence interval; ref.=reference group.
*P-value <.05, ** P-value <.01 and *** P-value <.001.
† Educational level: ‘High’: bachelor, master, doctoral or equivalent; ‘Middle’: upper secondary education, post-secondary non-tertiary education, short-cycle tertiary education; ‘Low’: no education, primary education, lower secondary education.

**Table S3** Associations between need, contextual and personal factors and online and offline media use for parenting information among participants of the CIKEO study (n=658).

|  | **Online and offline media use**  (‘yes’ n=181; 27.5%) |
| --- | --- |
|  | Full model OR (95% CI) |
| **Need factors**  *Questions or concerns related to parenting issues (more)* | **1.21 (1.12, 1.30)***** |
| **Personal factors** |  |
| *Age of the parent (in years)* | 0.99 (0.95, 1.04) |
| *Gender of the parent*  Female  Male | ref. 1.17 (0.50, 2.74) |
| *Educational level of the parent*†  High  Middle  Low | ref. **0.64 (0.43, 0.95)*** 0.34 (0.11, 1.00) |
| *Employment status of the parent*  Paid job  No paid job | ref. 1.00 (0.61, 1.63) |
| *Migration background of the parent*  No  Yes | ref. 1.25 (0.71, 2.21) |
| *Family situation*  Two-parent family  One-parent family | ref. 0.89 (0.34, 2.35) |
| *Age of the child (in years)* | 0.97 (0.87, 1.09) |
| *Gender of the child*  Girl  Boy | ref.  **0.65 (0.45, 0.94)*** |
| *Number of children in the household*  One child  Two children  More than two children | ref.  1.25 (0.79, 1.99) 1.56 (0.90, 2.71) |
| **Contextual factors** *Parenting information from personal social contacts*  No  Yes | ref. **2.19 (1.07, 4.49)*** |

Odds ratios and 95% confidence intervals were derived from the logistic regression analyses for online and offline media use for parenting information. P-values <.05 in bold.
Abbreviations: OR=odds ratio; CI=confidence interval; ref.=reference group.
*P-value <.05, ** P-value <.01 and *** P-value <.001.
† Educational level: ‘High’: bachelor, master, doctoral or equivalent; ‘Middle’: upper secondary education, post-secondary non-tertiary education, short-cycle tertiary education; ‘Low’: no education, primary education, lower secondary education.
